# Preclinical evaluation of tridecaptin M: *in vitro* and *in vivo* efficacy against colistin-resistant Gram-negative bacterial pathogens and pharmacokinetics

**DOI:** 10.1128/aac.01083-25

**Published:** 2025-09-11

**Authors:** Vrushali Raka, Manoj Jangra, Parminder Kaur, Rajneesh Dadwal, Shubhangi Kansal, Archana Angrup, Pallab Ray, Hemraj Nandanwar

**Affiliations:** 1 Clinical Microbiology & Antimicrobial Research Laboratory, CSIR-Institute of Microbial Technology 29748, Chandigarh, India; 2 Academy of Scientific & Innovative Research (AcSIR) 550336, Ghaziabad, Uttar Pradesh, India; 3 Department of Medical Microbiology, Post Graduate Institute of Medical Education and Research 685007, Chandigarh, India; Providence Portland Medical Center, Portland, Oregon, USA

**Keywords:** multidrug-resistant, gram-negative bacteria, colistin-resistant *Klebsiella pneumoniae*, pharmacokinetics, AMPs, tridecaptins, preclinical

## Abstract

The escalating threat of antimicrobial resistance (AMR), particularly among gram-negative pathogens, necessitates the development of novel therapeutic agents. Tridecaptins, a class of non-ribosomally synthesized lipopeptides with a novel mode of action, have garnered renewed interest in the fight against AMR. Our group previously identified tridecaptin M, a compound with a promising safety profile, prompting further investigation into its efficacy and preclinical characteristics. Here, we show that tridecaptin M exhibits potent activity against multidrug-resistant (MDR) *Klebsiella pneumoniae* and *Escherichia coli* without cross-resistance to colistin. It effectively inhibits biofilm formation and disrupts 50% of established biofilm at 10 µg/mL. Tridecaptin M demonstrates a favorable safety profile, as it does not inhibit the cardiac hERG channel and shows minimal interaction with cytochrome P450 enzymes, with no IC₅₀ detected up to 44.6 µg/mL. *In vivo* toxicity studies via subcutaneous administration confirm its safety up to 600 mg/kg, whereas intravenous administration reveals acute toxicity at ≥30  mg/kg, with biochemical evidence of skeletal muscle, cardiac, and hepatic involvement. In mouse infection models using a colistin-resistant MDR strain of *K. pneumoniae* reveal the *in vivo* potential of tridecaptin M and a dose-dependent efficacy at 10 mg/kg, 20 mg/kg, 50 mg/kg, and 100 mg/kg doses, showing a non-linear relationship. Tridecaptin M is metabolized by liver microsomes, with low clearance, and pharmacokinetic analysis in rats indicates favorable attributes, with a terminal half-life (T₁/₂) of 3.65 h intravenously and 8.81 h subcutaneously. Collectively, these data support the continued preclinical development of tridecaptin M as a promising candidate for treating severe gram-negative infections.

## INTRODUCTION

AMR, a critical global health threat with far-reaching implications for public health, politics, and the economy, jeopardizes the progress made in modern medicine (1). A 2019 study identifies six key pathogens, belonging to the ESKAPE group, as major contributors to AMR. Resistance to essential antibiotics like fluoroquinolones and β-lactams, crucial for treating severe infections, accounts for over 70% of AMR-related deaths across all pathogens (2). ESKAPE pathogens are frequently implicated in AMR and hospital-acquired infections, as confirmed by multiple studies (3). The World Health Organization’s 2024 priority pathogen list continues to include carbapenem-resistant *Klebsiella pneumoniae* and third-generation cephalosporin-resistant *Escherichia coli* as critical threats (4). The scarcity of effective treatments has led to the resurgence of colistin, a last-resort antibiotic, despite its significant neurotoxic and nephrotoxic risks (5). The COVID-19 pandemic exacerbated the AMR crisis, with many patients receiving unnecessary antibiotics, further driving resistance (6). The pandemic also underscored the importance of diagnostic testing, the catastrophic impact of the lack of effective treatments, and the need for incentives to develop new antimicrobials to prevent future outbreaks from escalating into pandemics (7). Hence, new antibiotics with novel modes of action are urgently needed.

**Fig 1:**
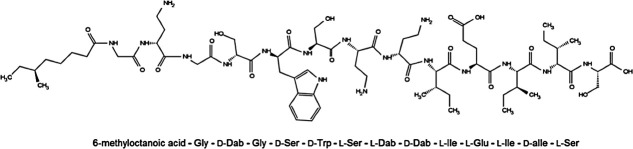
The chemical structure of tridecaptin M consists of N-terminal 6-methyl octonoic acid and 13-amino acid sequence.

Encouragingly, there has been an increase in early stage clinical candidates from 2019 to 2022. However, the number of newly approved drugs from 2020 to 2022 fell short of expectations. Most phase III antibiotics belong to established classes such as β-lactams, fluoroquinolones, macrolides, oxazolidinones, and topoisomerase inhibitors. Among the eight antibiotics approved since 2017, only two feature new chemical structures; the rest are derivatives of existing antibiotics. Peptides, such as antimicrobial peptides (AMPs) and non-ribosomal antibacterial peptides (NRAPs), are promising antimicrobial candidates (8) due to their diverse chemical structures and effectiveness in treating bacterial infections. Currently, several AMPs are in clinical trials, and some are already approved for use. For example, polymyxins treat various MDR gram-negative bacterial infections. Daptomycin, another AMP, is used to treat complex skin infections caused by gram-positive bacteria, particularly *Staphylococcus aureus* (9). NRAPs include vancomycin, bacitracin, and cyclosporin, which are being used clinically (10), and lugdunin is under development against gram-positive pathogens (11). Among NRAPs, a class of linear cationic antimicrobial peptides known as tridecaptins has gained attention due to their effectiveness against gram-negative pathogens. Tridecaptins bind to lipid II, a cell wall precursor, and disrupt the proton-motive force of bacterial membranes, leading to cell death (12). Despite their significant activity against antibiotic-resistant bacteria, the total synthesis of tridecaptin presents challenges, including the need for costly chiral moieties and multiple synthesis steps (13, 14). In 2019, our group reported tridecaptin M (Fig. 1), a new member of this family, with *in vitro* and *in vivo* activity against MDR and colistin-resistant bacteria (15, 16). Tridecaptin M was non-hemolytic and non-cytotoxic at concentrations well above its MIC in bacteria (16). Furthermore, it showed well tolerability in mice. Tridecaptin M also shows synergistic activity with rifampicin and vancomycin against *Acinetobacter baumannii* (17).

This study aims to further investigate the preclinical potential of tridecaptin M as an antimicrobial agent against colistin- and carbapenem-resistant *Enterobacteriaceae* (CRE). Here, we demonstrate the *in vitro* activity of tridecaptin M against colistin-resistant clinical pathogens, with effective MICs across all tested strains. We also assessed the compound’s efficacy in eradicating preformed biofilms. *In vitro* drug kinetics and toxicity, *in vivo* rat pharmacokinetics, and mouse toxicity studies, along with *in vivo* efficacy in various murine infection models, underscore the potential of tridecaptin M as a promising antibacterial agent against gram-negative pathogens.

## RESULTS

### Antimicrobial activity of tridecaptin M against clinical isolates

To evaluate the potency of tridecaptin M, a total of 121 *K*. *pneumoniae* and 64 *E. coli* bacterial clinical isolates from the Post-Graduate Institute of Medical Education and Research (PGIMER), Chandigarh, were tested for antimicrobial susceptibility using the microbroth dilution method. The isolates were MDR with % resistance against *K. pneumoniae* and *E. coli,* respectively, for each antibiotic as amikacin R (67.7/45.3), cefotaxime R (36.3/25), cefepime R (58.6/32.2), gentamicin R (74.3/57.8), imipenem R (81.8/53.1), cefoperazone R (35.5/28.8), meropenem R (58.6/37.5), and tigecycline R (4.1/0). These profiles for each strain were determined by the disk diffusion method when samples were collected from patients. We evaluated the minimum inhibitory concentrations (MICs) of tridecaptin M and colistin against each bacterial strain. Colistin, a last-resort antibiotic used to treat multidrug-resistant (MDR) infections, showed MIC values ranging from ≤0.25 µg/mL to ≥32 µg/mL across both species. Since tridecaptin M is active against colistin-resistant strains, and both antibiotics belong to the class of non-ribosomal peptides (NRPs). Colistin was included in the study to assess potential cross-resistance. Based on EUCAST and CLSI breakpoints, colistin resistance (MIC >2 µg/mL) (18, 19) was observed in 49 of 121 *K*. *pneumoniae* and 12 of 64 *E. coli* isolates. Tridecaptin M (reported MIC = 4 µg/mL) (16) exhibited MIC values between 2 and 8 µg/mL for all isolates, regardless of colistin susceptibility status. The MIC₅₀ and MIC₉₀ (the lowest concentration of drug that inhibits 50% and 90% of the isolates, respectively) values for colistin-sensitive and colistin-resistant strains were comparable, indicating that tridecaptin M retains activity against colistin-resistant isolates and does not exhibit cross-resistance (Table 1).

**TABLE 1:** MIC distribution of tridecaptin M and colistin against clinical strains^*a*^

Tridecaptin M								Colistin	
*K. pneumoniae* (*K. P*)				*E. coli*				(*K. P*)	*E. coli*
(Number of isolates)	MIC range µg/mL	MIC_50_ µg/mL	MIC_90_ µg/mL		MIC range µg/mL	MIC_50_ µg/mL	MIC_90_ µg/mL	MIC range µg/mL	MIC range µg/mL
Col-S (72)	2-8	4	8	Col-S (52)	1-8	4	4	0.25-2	0.25-1
Col-R (49)	2-8	8	8	Col-R (12)	1-8	4	8	4 – > 32	4 – > 32
Std. QC (ATCC 700603)	4	–^*b*^	–	Std. QC (ATCC 25922)	4	–	–	0.5	0.25

^*a*^ Col-S (colistin sensitive), Col-R (colistin resistant), >32 µg/mL, as this was the highest concentration tested for colistin. MIC_50_ and MIC_90_ are the lowest concentrations of compound (tridecaptin M) at which 50% and 90% of the clinical isolates are inhibited. Standard QC strains used are *K. pneumoniae* ATCC 700603, and *E. coli* 25922. Colistin MIC value greater than 2 µg/mL was considered resistant (18).

^*b*^ –, not determined.

### Tridecaptin M inhibits and disrupts pre-formed biofilms

**Fig 2:**
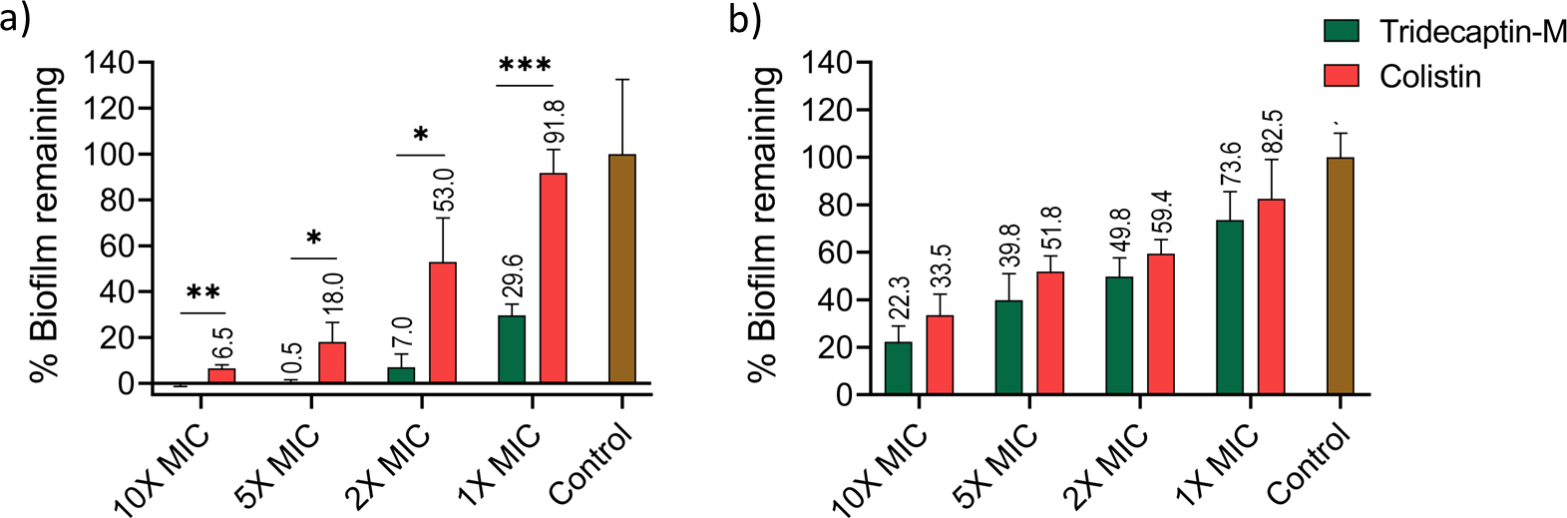
Effect of tridecaptin-M on the biofilm compared with colistin. The crystal violet dye assessed the biomass of *K. pneumoniae* ATCC 700603. Tridecaptin M and colistin (MIC 4 µg/mL and 0.5 µg/mL, respectively) at 1×, 2×, 5×, and 10× MIC concentrations were used in biofilm inhibition and eradication assays. (**A**) Biofilm inhibition after 48 h incubation, calculated based on the absorbance of crystal violet at 590 nm and represented as % biofilm remaining. (**B**) Biofilm disruption of preformed biofilm after treatment for 24 h, represented as the percentage of biofilm remaining. The results presented here represent the average ± SD of three independent measurements. Statistical significance was determined as significant when *P < 0.05, **P < 0.01, and highly significant as ***P < 0.001.

A common complication of nosocomial infections is their ability to form biofilms, which play a key role in horizontal gene transfer and contribute significantly to the development of multidrug resistance. These biofilms are difficult to eradicate and are often associated with increased morbidity (20). In this study, we evaluated the ability of tridecaptin M to both inhibit biofilm formation and eradicate preformed biofilms. Using the crystal violet (CV) assay, we observed that tridecaptin M was significantly more effective at inhibiting biofilm formation than colistin. At 5× MIC, tridecaptin M nearly completely inhibited the biofilm formation (Fig. 2a). When treating preformed *K. pneumoniae* biofilms with tridecaptin M, partial disruption was achieved, 51%, 61%, and 78% at 2×, 5×, and 10× MIC, respectively, with colistin also demonstrating similar levels of partial disruption in this assay (Fig. 2b).

### *In vitro* drug kinetics, toxicity studies, and *in vivo* pharmacokinetics of tridecaptin M

**Fig 3:**
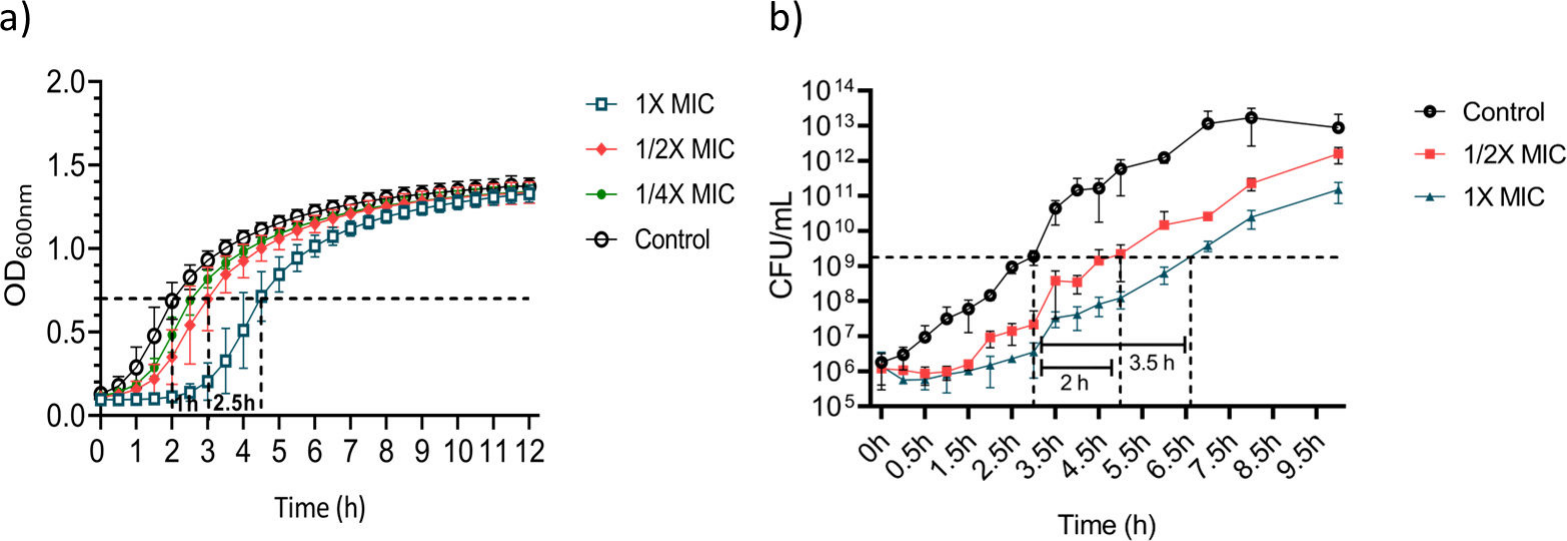
(a) Post-antibiotic effect (PAE) of tridecaptin M at 1/2 MIC, 1/4 MIC, and 1 MIC in *K. pneumoniae* ATCC 700603 by the turbidimetry-based system. Time 0 h corresponds to the growth monitoring after antibiotic exposure for 2 h and cell resuspension in a fresh medium, with growth monitoring for 12 h. The data represented is the average ± SD value of triplicate. (b) PAE of tridecaptin M at 1/2 MIC and 1 MIC by the CFU/mL based method. Experimental parameters were the same as those of the turbidometry-based method. The data represented is the average ± SD value of triplicate.

We investigated the *in vitro* drug kinetics of tridecaptin M, which is critical to the preclinical development of drugs. We first tested the post-antibiotic effect (PAE) of tridecaptin M in *K. pneumoniae.* PAE refers to the sustained suppression of bacterial growth following brief exposure to an antibiotic. This effect is crucial, as it allows for longer intervals between doses, enhancing the therapeutic efficiency of the antibiotic. A PAE of greater than 0.5 h indicates a significant impact on bacterial growth by the tested compound. For tridecaptin M, the PAE durations were observed to be approximately 1 h at 0.5× MIC and 2.5 h at 1× MIC, as determined using a turbidometric method (Fig. 3a). To strengthen these findings and provide a more reliable correlation, we performed a complementary experiment by quantifying viable bacterial counts (CFU/mL) following exposure to tridecaptin M (Fig. 3b). A slight decrease in CFU was noted at 15 min compared with time 0, likely due to the compound having already caused irreversible damage to bacterial cells during the initial exposure period, resulting in continued cell death even after its removal. Compared with the untreated control, a delay in bacterial regrowth (lag phase) of approximately 2 h at 0.5× MIC and 3.5 h at 1× MIC was observed, further supporting the presence of a dose-dependent PAE.

**Fig 4:**
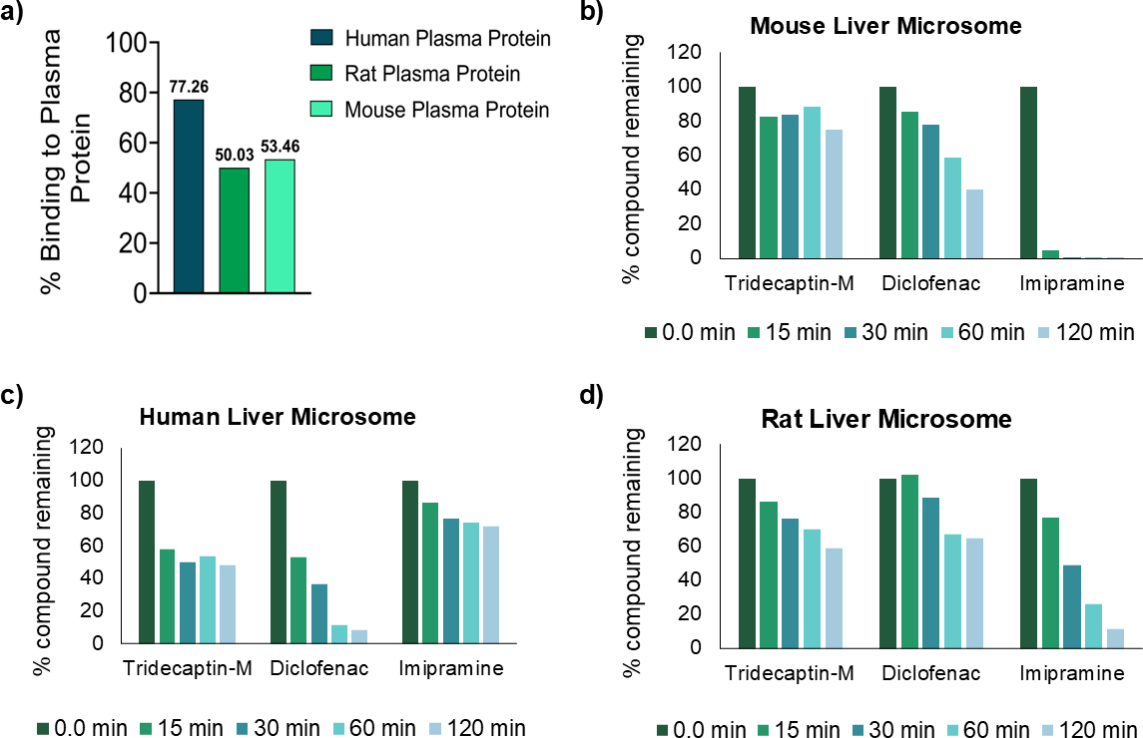
(a) Plasma protein binding of tridecaptin M with mouse, rat, and human plasma using the RED insert device method, and the free compound was quantified using LC-MS/MS analysis. The experiment was performed in duplicates. (b) Microsomal stability assay performed with mouse, (c) human, and (d) rat liver microsomes for up to 120 min shows low clearance. Positive control diclofenac and imipramine, known compounds metabolized by liver microsomes, were included in the study. The experiment was performed in duplicates.

Another important factor responsible for the *in vivo* efficacy of successful drugs is plasma protein binding. The rapid equilibrium dialysis method was employed to determine the concentration of free drug available. The study revealed that tridecaptin M binds to plasma proteins in humans, mice, and rats at rates of 77.26%, 50.03%, and 53.46%, respectively (Fig. 4a). *In vivo* efficacy is determined by the absolute free drug concentration at the target site, not merely the free fraction; therefore, drugs with >98% bound to plasma protein are also successful therapeutic candidates (21). We then assessed the hepatic microsomal stability and clearance of tridecaptin M using liver microsomes. Understanding a drug’s metabolic stability is critical for predicting its clearance rate and potential drug-drug interactions, especially involving liver enzymes. In our study, tridecaptin M demonstrated low clearance rates in human (17.6 µL/min/mg), mouse (<11 µL/min/mg), and rat liver microsomes (<11 µL/min/mg) (Table S1). The concentration of tridecaptin M was measured over 120 min and compared with baseline levels at 0 min, showing minimal degradation over time (Fig. 4b through d). In contrast, the positive control drugs, diclofenac in humans and imipramine in rodents, were rapidly metabolized in the presence of liver microsomes with cofactors, confirming the assay’s reliability. Additionally, tests without liver enzyme cofactors confirmed that the absence of enzymatic activity resulted in no significant change in compound concentration after 120 min (Fig. S1a through c).

The potential cardiotoxicity of tridecaptin M was assessed by evaluating its effect on the heart’s potassium hERG channel, a common target for drug-induced QT interval prolongation. The study utilized the patch-clamp technique on HEK293 cells expressing hERG channels, with Quinidine serving as a positive control, which showed the expected inhibitory effects. Tridecaptin M exhibited no significant inhibition of the hERG channel at concentrations up to 30 µM (44.6 µg/mL), suggesting a low risk of inducing cardiac arrhythmias (Table 2).

**TABLE 2:** Cardiotoxicity assessment with hERG potassium heart channel inhibition in the presence of tridecaptin M and quinidine (positive control)

Tridecaptin M (µM)	Mean current (A)	Mean % inhibition	SD	Quinidine (µM)	Mean current (A)	Mean % inhibition	SD
30	4.68E-10	**−6.44^*a*^**	6.48	10	2.24E-11	**93.19**	0.35
10	4.34E-10	**1.37**	2.21	3	1.19E-10	**63.87**	0.53
3	4.58E-10	**−4.07**	3.47	1	1.29E-10	**60.83**	1.69
1	4.64E-10	**−5.54**	5.23	0.3	1.75E-10	**46.76**	1.67
0.3	4.66E-10	**−5.86**	3.52	0.1	2.32E-10	**29.45**	0.46
0.1	4.41E-10	**−0.37**	2.28	0.03	2.86E-10	**13.06**	2.87
**Control**	4.40E-10	**0.00**	2.41	**Control**	3.29E-10	**0.00**	5.62

^*a*^ Bold value indicates % inhibition with increasing concentration of each compound.

Next, we performed a CYP enzyme inhibition assay using recombinant isoforms (CYP1A2, CYP2C9, CYP2C19, CYP3A4, and CYP2D6) to assess the potential for drug-drug interactions. Tridecaptin M displayed minimal effect (CYP 2D6, 2C9, 2C19, and 3A4) or no inhibitory effects (CYP 1A2) across tested concentrations up to 30 µM (44.6 µg/mL), with none of the CYP isoforms showing 50% inhibition, meaning it interacts weakly with the CYP enzymes, leading to minimal inhibition even at high concentrations, and IC50 could not be determined (Tables S2 to S6). The inclusion of known inhibitors validated the assay’s sensitivity, with observed IC50 values for positive controls noted along with the highest concentrations of tridecaptin M (30 µM) tested and percent inhibition achieved at this highest concentration (Table 3). We also tested the mutagenicity of tridecaptin M with the AMES test based on the potential of the compound to reverse selective growth mutations in several strains of *Salmonella typhimurium* in the presence and absence of S9 (liver microsomal enzyme). The compound did not show any mutagenic potential at 3.16, 1.00, 0.32, and 0.10 µg/plate concentrations; the positive controls showed a mutagenic response as per the acceptance criteria. Tridecaptin M at 10 µg/plate and above could not be tested as these concentrations were found to be cytotoxic to *Salmonella typhimurium* strains (Table S7).

**TABLE 3:** Major CYP enzyme inhibition by Tridecaptin-M and respective positive controls^*a*^

Highest tested concentration (µM)	Test/Positive control items	IC_50_ (µM)				
		CYP 1 A2	CYP 2 C9	CYP 2 C19	CYP 2D6	CYP 3 A4
**Tridecaptin-M**	**30**	**>30**	**>30**	**>30**	**>30**	**>30**
Furafylline (Positive control)	30	1.43	–^*c*^	–	–	–
Sulfaphenazole (Positive control)	3	–	0.71	–	–	–
Tranylcypromine (Positive control)	50	–	–	1.57	–	–
Quinidine (Positive control)	1	–	–	–	0.003	–
Ketoconazole (Positive control)	10	–	–	–	–	0.02
**% inhibition by tridecaptin-M**	**30^*b*^**	**≤ 0**	**25.6**	**11.65**	**13.11**	**26.59**

^*a*^ IC_50_ = Inhibitory concentration at which 50% inhibition occurs. IC_50_ was not achieved for tridecaptin M up to 30 µM concentration tested. At the highest concentration of 30 µM of tridecaptin M, the percent inhibition observed for each CYP tested is noted at the bottom row of the table.

^*b*^ Bold values indicates % inhibition of each CYP enzyme at highest tridecaptin M concentration tested.

^*c*^ –, not determined.

Pharmacokinetics (PK) of tridecaptin M in Sprague–Dawley rats, followed by a subcutaneous (SC) administration and an intravenous (IV) bolus administration at 5 mg/kg body weight (b.w), was performed, and plasma pharmacokinetic parameters were determined by non-compartmental analysis using Phoenix WinNonlin software. For subcutaneous PK, Cmax achieved was 9.15 µg/mL, whereas in IV PK, tridecaptin M displayed a Cmax of 38.5 µg/mL, about 10 times higher than the MIC value, as it is directly administered into the blood. The plasma exposure (AUClast) and mean residence time were found to be 14.05 h*µg/mL and 8.81 h for the SC route and 94.61 h*µg/mL and 2.69 h for the IV route, respectively. The terminal half-life (T1/2) of tridecaptin M was 8.56 h and 3.65 h with a clearance rate of 4.95 mL/min/kg and 1 mL/min/kg in SC and IV PK, respectively (Table 4). At a 5 mg/kg dose, the concentration was maintained above the MIC value of tridecaptin M administered intravenously for more than the calculated T1/2 (Fig. S1d). Tridecaptin M shows favorable pharmacokinetics via the IV route. Although IV administration provides rapid and high plasma concentrations suitable for immediate therapeutic effect, SC administration offers a longer mean residence time, supporting its potential for sustained exposure and the feasibility of alternative delivery methods at high concentrations for use in clinical settings. The ability of IV dosing to maintain concentrations above the MIC beyond its half-life suggests a strong pharmacodynamic window and the possibility of effective dosing in clinical applications.

**TABLE 4:** Intravenous pharmacokinetics (PK) plasma parameters for tridecaptin M in male Sprague–Dawley rats^*a*^

PK parameters	Mean plasma PK parameters Intravenous	Mean plasma PK parameters Subcutaneous
Dose (mg/kg b.w.)	5	5
C_max_ (µg/mL)	43.2 ± 6.02	9.15 ± 1.7
AUC_last_ (h*µg/mL)	94.61 ± 44.98	14.05 ± 2.09
T_1/2_ (h)	3.65 ± 0.56	8.56 ± 1.60
MRT_last_ (h)	2.69 ± 1.02	8.81 ± 2.1
CL (mL/min/kg)	1.00 ± 0.33	4.95 ± 0.24
Vss (L/kg)	0.16 ± 0.04	2.63 ± 0.45

^*a*^ C_max_ = maximum serum concentration, AUC = Area under the curve, T_1/2_ = Half-life, MRT = Mean residence time, CL = clearance rate, Vss = volume of distribution. PK parameters were determined by non-compartmental analysis using Phoenix WinNonlin software.

Tridecaptin M has demonstrated promising results in various *in vitro* assays, including low interactions with CYP enzymes, the hERG channel activity, plasma protein binding, and a notably low clearance rate by liver microsomes. These attributes are favorable from a regulatory perspective and support the continued development of tridecaptin M as a potential antibacterial agent, with reduced metabolism-related drug interactions.

### *In vivo* tolerability and safety profile of tridecaptin-M

The previous study by our group identified tridecaptin-M to be safe for mammalian cell lines, and no hemolysis was observed at the tested concentrations. Moreover, the compound demonstrated good tolerance in acute toxicity studies involving mice, which were given doses of 12 mg/kg every 2 h for up to 12 h, resulting in a total accumulated dose of 72 mg/kg of tridecaptin M. In contrast, all mice in the colistin group died within 24 h, as doses exceeding 36 mg/kg subcutaneously are known to be lethal to mice (16). In the current study, we administered tridecaptin M and colistin at a dose of 15 mg/kg/day, divided into two subcutaneous injections daily for 14 days. This dosage was chosen to replicate the typical 2-week dose regimen of colistin in a clinical setting and compare tridecaptin M with colistin at the same concentration. Above this concentration, signs of toxicity may begin to appear in the case of colistin (22, 23). The effects of tridecaptin M and colistin on the liver and kidneys of mice were compared with a control group through histopathological staining, imaging, and serum biochemical liver and kidney function tests. The biochemical parameters (Table 5) were comparable with those of the control group (2.5% DMSO). SGOT levels were significantly elevated in all experimental groups, including the control, compared with the reference ranges reported in the literature (24–26). This elevation may be attributed to the presence of 2.5% DMSO used as a vehicle in both the control and tridecaptin M groups. However, since DMSO was not used in the colistin group, the observed increase in SGOT levels in this case may be a direct effect of colistin administration. Histopathological analysis revealed no signs of toxicity in the liver and kidneys of both tridecaptin M and colistin groups (Fig. S2a).

**TABLE 5:** Biochemical kidney serum parameters for repeat dose toxicity for 14 days at 15 mg/kg/day divided into two doses

Serum parameter	Colistin Mean ± SD	Tridecaptin M Mean ± SD	Control Mean ± SD	Reference range (24–26)
Kidney (renal parameters)				
Calcium (mg%)	9.36 ± 0.99	7.76 ± 2.19	8.66 ± 0.39	**6.6–9.8^*a*^**
BUN (mg%)	35.33 ± 4.84	35.33 ± 11.43	33.33 ± 3.01	**18–29**
Creatinine (mg%)	0.49 ± 0.07	0.41 ± 0.026	0.46 ± 0.04	**0.2–0.7**
ALP (U/L)	79 ± 26.16	77.33 ± 27.47	103.66 ± 6.62	**62–209**
Phosphorous (mg%)	9.23 ± 2.21	7.36 ± 1.27	8.13 ± 0.80	**5.1–10.2**
Uric acid (mg%)	5.96 ± 1.28	4.6 ± 1.89	5.73 ± 0.87	**1.7–5.4**
Liver (hepatic parameters)				
BBilirubin (mg/dL)	0.30 ± 0.09	0.31 ± 0.06	0.33 ± 0.09	**0.1–0.9**
SGOT (Units/L)	947.33 ± 443.22	657.33 ± 141.06	834.67 ± 241.05	**59–247**
SGPT (Units/L)	135.67 ± 69.26	103.67 ± 40.70	107.00 ± 32.91	**28–132**
Proteins (g/dL)	7.27 ± 1.23	6.13 ± 1.06	6.43 ± 0.37	**4.4–7.6**
Albumin (g/dL)	3.37 ± 0.46	2.60 ± 0.47	3.03 ± 0.23	**2.7–4.9**
Globulin (g/dL)	3.9 ± 0.89	3.53 ± 0.83	3.40 ± 0.28	**–^*b*^**

^*a*^ Bold indicates reference range for each biochemical parameter.

^*b*^ –, not available.

To determine the maximum tolerated dose (MTD) of tridecaptin M, an acute toxicity study was performed. Mice received a single subcutaneous dose of tridecaptin M at 100 mg/kg, 200 mg/kg, 400 mg/kg, and 600 mg/kg. None of the groups displayed any signs of lethargy or toxicity. The mice were monitored for 3 days, after which they were euthanized, and major organs, including the heart, kidney, liver, brain, and spleen, were harvested for histopathological analysis. No signs of toxicity or pathological changes were observed in any of the treated groups compared with the vehicle control group (Fig. S2b). Serum samples from the mice were analyzed biochemically, revealing no significant differences in most parameters, except for fluctuations in serum glutamic pyruvate transaminase (SGPT) and serum glutamic oxaloacetic transaminase (SGOT) levels. These values were elevated in both the control (100% DMSO) and treatment groups due to the DMSO concentration used. However, in TF-2, the levels were even higher than in the control, suggesting a potential toxic effect on the liver at higher doses (Table 6).

**TABLE 6:** Biochemical serum tests for acute toxicity of tridecaptin-M at 600 mg/kg administered subcutaneously^*a*^

Test parameter	CM-1	CM-2	CF-1	CF-2	TM-1	TM-2	TF-1	TF-2	Ref. range (24–26)
Urea (mg%)	34	30	30	30	30	30	38	30	**–^*c*^**
Creatinine (mg%)	0.40	0.40	0.44	0.44	0.40	0.40	0.40	0.4	**0.2–0.7**
Uric acid (mg%)	2.0	2.0	3.2	2.2	2.2	3.6	2.0	2.6	**1.7–5.4**
Bilirubin (mg/dl)	0.44	0.48	0.44	0.42	0.42	0.44	0.4	0.5	**0.1–0.9**
SGOT (Units/L)	412	478	474	492	566	508	536	**818^*b*^**	**59–247**
SGPT (Units/L)	66	70	84	84	80	80	82	**112**	**28–132**
Triglycerides (mg%)	158	124	118	108	158	100	114	98	**62–155**

^*a*^ CM = control male mice, CF = control female mice, TM = Tridecaptin M administered male mice, TF = Tridecaptin M administered female mice.

^*b*^ Reference range for biochemical parameter and elevated values compared to references are highlighted in bold.

^*c*^ –, not available.

Furthermore, MTD was evaluated for tridecaptin M administered intravenously (i.v.) in mice. Administration of tridecaptin M at 100 mg/kg was immediately lethal, similar to the effect observed with colistin at 18 mg/kg intravenously. A dose of 50 mg/kg of tridecaptin M also resulted in mortality; however, mice treated with 25 mg/kg or less of tridecaptin M showed no visible signs of toxicity or mortality. Therefore, next, we administered the compound intravenously at lower dose intervals of 5 mg/kg post-25 mg/kg dose and observed every 1 h post-administration. In the 30 mg/kg dose group, mice survived for 6–10 h post-administration but displayed abnormal motor behavior, including dragging of limbs. Similar clinical signs were observed in the 35 mg/kg and 40 mg/kg tridecaptin M groups, with the 40 mg/kg group exhibiting a rapid onset of toxicity, resulting in mortality within 2 h. Despite these clinical symptoms, histopathological analysis of major organs, including the liver, kidney, heart, and brain, revealed no significant morphological alterations compared with the control group (Fig. S2c). However, biochemical analysis indicated elevated serum levels of SGOT, BUN (Blood Urea Nitrogen), CPK (Creatine Phosphokinase), and CPK-MB, along with slightly reduced lipase (Table 7). These biochemical changes, together with the observed motor impairments, suggest possible involvement of skeletal and cardiac muscle (probably no QT prolongation, as no hERG channel inhibition was observed) and neurotoxicity. The elevated enzyme levels may result from cellular stress or membrane disruption, rather than from irreversible tissue damage. Further investigation using more sensitive molecular and ultrastructural methods is required to elucidate the precise mechanisms underlying this systemic toxicity.

**TABLE 7:** Biochemical kidney serum parameters for intravenously administered tridecaptin M toxicity^*a*^

Test	Control	Control (20% DMSO)	20 /kg	25 /kg	40 /kg	Reference range (24–26)
SGOT (Units/L)	164	208	244	**872^*b*^**	**784**	**59–247**
SGPT (Units/L)	56	68	68	**208**	88	**28–132**
BUN (mg%)	32	44	96	112	24	**18–29**
CPK (U/L)	400	808	448	712	**24,472**	**68–1070**
LDH (U/L)	1412	2340	2120	3896	3280	**1105–3993**
Glucose (mg/dL)	256	300	344	248	152	**75–128**
Lipase (U/L)	1204	1296	1016	972	**368**	**–^*c*^**
Amylase (U/L)	372	3696	3800	**5144**	**1560**	**1691–3615**
CPK_MB (U/L)	384	740	420	**952**	**1408**	**–**

^*a*^ Serum was isolated from blood at 24 h after administration of the drug to each group, except 40 mg/kg, where the death of mice occurred within 2 h after administration, and immediately, as the visible movement of mice stopped, the blood was collected. The serum from each mouse was pooled together for each group for biochemical analysis.

^*b*^ Reference range for each biochemical parameter and elevated values compared to reference range are highlighted in bold.

^*c*^ –, not available.

### *In vivo* efficacy of tridecaptin M in murine infection models

**Fig 5:**
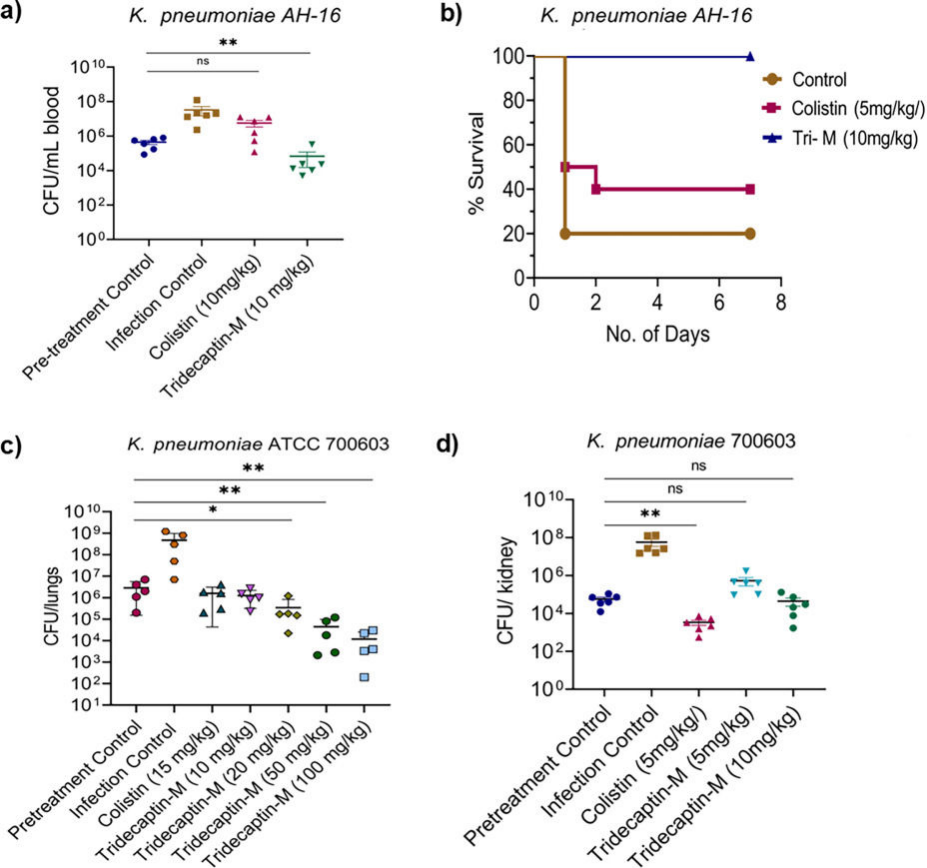
(a) *K. pneumoniae* AH-16 colistin-resistant strain was used to establish a blood infection model in Balb/c mice (*n* = 6). Single-dose treatment of tridecaptin-M and colistin at 10 mg/kg was administered. At 20 h, post-treatment, CFU was determined. (b) Sepsis model was induced in Balb/c mice (*n* = 10) with a high load of *K. pneumoniae* AH-16 (10^8^ CFU/mL) in 5% mucin. Mice were treated with tridecaptin-M (10 mg/kg) and colistin (5 mg/kg to adhere to a collective of 15 mg/kg/day dosing limit for colistin) in three doses at 2 h, 12 h, and 24 h post-infection. Mice were observed for 7 days, and the percent survival is represented by a Kaplan-Meier survival plot. (c) A lung infection model to mimic pneumonia was established using *K. pneumoniae* ATCC 700603, a colistin-sensitive strain in Balb/c mice (*n* = 5). Treatment with varying doses of tridecaptin-M 10 mg/kg, 20 mg/kg, 50 mg/kg, 100 mg/kg, and colistin at 15 mg/kg (maximum per day dose) was administered. At 20 h post-treatment, mice were euthanized to harvest lungs, and the CFU was determined. Treatment for these three infection models was administered subcutaneously. (d) Balb/c mice (*n* = 6) were given an infection of *K. pneumoniae* ATCC 700603 intraperitoneally, and colistin and tridecaptin M were administered intravenously in a single dose at 4 h post-infection. At 20 h post-infection, the kidney was harvested, and the CFU/kidney was determined. CFU results were considered highly significant when **P < 0.01, ***P < 0.001, and ****P < 0.0001.

In a previous study, tridecaptin M demonstrated efficacy in a murine thigh infection model of *K. pneumoniae,* showing activity against both sensitive and colistin-resistant strains (16). In this study, we expanded on this by establishing multiple murine infection models, including the blood septicemia model and peritonitis survival using the colistin-resistant *K. pneumoniae* AH-16 clinical isolate, and lung infection with colistin-sensitive *K. pneumoniae* ATCC 700603. Tridecaptin M exhibited potent activity at a dose of 10 mg/kg administered subcutaneously, resulting in a reduction of bacterial load in blood compared with the control. (Fig. 5a). In the peritonitis survival model, tridecaptin M treatment led to 100% survival of the mice at the end of 7 days, compared with 40% survival in the colistin-treated group after three doses of 10 mg/kg tridecaptin M and 5 mg/kg colistin, respectively, and 20% survival in the untreated control group against colistin-resistant strain (Fig. 5b). Colistin was administered at a dose of 5 mg/kg in the survival model to adhere to the recommended maximum daily dose of 15 mg/kg/day in total three doses. To study the dose-dependent effect of tridecaptin M, we used multiple concentrations in comparison to colistin at 15 mg/kg (maximum single dose) in the lung infection model. There was a decrease in bacterial burden with increasing concentration of tridecaptin M, but the decrease was not exactly proportional to increasing dose (Fig. 5c).

We also studied the efficacy of tridecaptin M administered intravenously (i.v.) in a mouse peritonitis model with colistin-sensitive *K. pneumoniae* ATCC 700603. Tridecaptin M, administered as a single dose at 5 mg/kg and 10 mg/kg, exhibited efficacy in the kidney. However, colistin exhibited superior performance, likely attributable to its lower MIC values and more rapid bactericidal activity compared with tridecaptin M (Fig. 5d). Collectively, these results suggest that tridecaptin M can be further developed as a therapeutic drug against MDR and colistin-resistant *Enterobacteriaceae*.

## DISCUSSION

Antibiotic resistance remains a significant challenge in modern medicine, driven largely by the overuse and misuse of antibiotics, leading to the emergence of resistant bacterial strains (27). Gram-negative bacteria, in particular, are a major contributor to mortality among antimicrobial-resistant pathogens. The rise in MDR gram-negative pathogens has created an urgent need for new antibiotics with novel mechanisms of action. Tridecaptin M, a non-ribosomal lipopeptide, exhibits *in vitro* activity against these MDR pathogens, including colistin-resistant strains. Despite the encouraging findings of tridecaptin M, several critical considerations must be addressed before clinical translation can be envisioned (28). In the current study, we explored the preclinical safety and efficacy of Tridecaptin M, an antibiotic with potent activity against colistin-resistant and carbapenem-resistant *Enterobacteriaceae* (CRE) pathogens. MIC values of Tridecaptin M were comparable in colistin-sensitive and -resistant isolates. This suggests that Tridecaptin M may circumvent existing resistance mechanisms and does not share cross-resistance with colistin, despite both being non-ribosomal peptides. CYP enzyme inhibition suggests the likelihood of drug–drug interactions at even lower concentrations, but inhibition is low, therefore supporting possible compound use at therapeutically relevant exposures.

The pharmacokinetic (PK) profile of Tridecaptin M in Sprague–Dawley rats provides initial insights into its systemic behavior. Following intravenous (IV) administration at 5  mg/kg, plasma concentrations exceeded the MIC for extended durations, supporting its pharmacodynamic potential. However, the relatively short terminal half-life (3.65  h) and Cmax raise questions about the feasibility of maintaining therapeutic levels *in vivo* without frequent dosing. Although subcutaneous (SC) administration is not a clinically viable route for this compound, SC PK data remain valuable for assessing absorption kinetics and guiding preclinical dosing strategies. SC dosing demonstrated a longer mean residence time (8.81 h) and a terminal half-life of 8.56 h, reflecting slower absorption and prolonged systemic exposure. These data suggest that formulation optimization will be critical to improving systemic exposure and reducing dosing frequency, potentially via liposomal encapsulation, PEGylation, or depot-based delivery systems.

Although tridecaptin M exhibits bactericidal activity, the observed reduction in bacterial load in single-dose *in vivo* experiments was modest. This may be explained by its pharmacokinetic profile, specifically its half-life (T₁/₂) and limited free drug availability, as bacterial load was assessed 20 h post-treatment. In contrast, a survival model employing three doses within a 24 h period demonstrated significantly improved outcomes, indicating that multiple dosing is necessary to achieve a more pronounced reduction in bacterial burden *in vivo*. Moreover, subcutaneous administration of tridecaptin M appeared to be well-tolerated; however, *in vivo* intravenous tolerability studies at doses ≥ 30  mg/kg revealed transient neurotoxicity, including limb-dragging and abnormal locomotor activity, with biochemical serum analysis indicating multiple toxicity parameters involved. Although these findings may be manageable through careful dose optimization, they underscore the need for rigorous toxicological evaluation and may reflect off-target effects or accumulation in peripheral tissues. One additional limitation lies in the modest MIC values observed (2-8  µg/mL), which, although consistent, are higher than those of many standard-of-care antibiotics. Whether these concentrations can be safely achieved and maintained in humans remains a central question for development. Furthermore, the lack of oral bioavailability, a common limitation for lipopeptides, restricts their use to parenteral administration unless structural modifications or delivery platforms are introduced. Strategies such as prodrug design or salt formation may enhance bioavailability while minimizing toxicity.

In conclusion, tridecaptin M displays multiple favorable attributes, including broad-spectrum activity against MDR strains, low potential for drug–drug interactions, and a manageable PK profile. However, the compound is not without limitations; the need for IV delivery, relatively high MICs, and potential toxicity at relatively low doses need to be carefully considered. Further studies should focus on optimizing the IV formulation, delivery, investigating alternative clearance mechanisms, tissue accumulation, and identifying toxicological mechanisms.

## MATERIALS AND METHODS

### Bacterial strains, media, and growth conditions

The standard ATCC strains, *K. pneumoniae* ATCC 700603, *E. coli* ATCC 25992, and *A. baumannii* ATCC 19606, *Paenibacillus jamilae* M-152 lab isolated strain, and *K. pneumoniae* AH-16 clinical isolate from Apollo Hospital, Chennai, and 244 isolates from PGIMER, Chandigarh were used in this study. Muller-Hinton Broth, Cation-adjusted MHB, and M-14 lab-optimized media were used for the growth of culture and were grown at 37°C, 200 rpm (except M-152 which was grown at 30°C) unless stated otherwise.

### Production and purification of tridecaptin-M

Tridecaptin-M was naturally produced by the bacteria *Paenibacillus jamilae* in 2L flasks in lab-optimized media M- 14 (Starch 20 g/L, NH_4_Cl_2_ 10 g/L, Casein enzyme hydrolysate 17.5 g/L, MgCl_2_ 20 mM, KH_2_PO_4_ 1.5 g/L, K_2_HPO_4_ 0.5 g/L, and isoleucine 0.5 g/L). Inoculum was prepared in 100 mL M14 media, and the log phase culture was grown at 30°C and was transferred to 2 L flasks containing 1000 mL sterile M14 media and allowed to grow for the next 24 h at 30°C, 200 rpm. The cultures were harvested by centrifugation, and further purification steps were followed as previously described (16). In brief, the crude extract was prepared using Diaion HP-20 resin, followed by cation-exchange chromatography using SP-Sepharose column and 1M NaCl as eluent. Furthermore, after desalting, reverse-phase HPLC with a C18 column was used to purify the compound. Finally, the purified compound was lyophilized to obtain a pure (≥ 95%) powder form of tridecaptin-M (Fig. S3).

### Activity check: minimum inhibitory concentration (MIC)

The MIC for all isolates was determined using the broth microdilution method. This involved preparing CA-MHB medium and serially diluting antibiotic solutions by a factor of two. The test organism was adjusted to a concentration of ≈5 × 10^5^ CFU/mL. In a sterile 96-well microtiter plate, 100 µL of CA-MHB, along with 100 µL of each antibiotic, was added, and serial dilutions were made. A 100 µL suspension of the organism was added to each well and mixed, resulting in a total reaction volume of 200 µL per well. The plate was then incubated at 37°C for 16–18 h. The MIC was identified as the lowest concentration of antibiotic that prevented any visible growth of the organism (16). MIC_50_ and MIC_90_ represented the lowest concentrations at which 50% and 90%, respectively, of the isolates were inhibited (29).

### Biofilm inhibition and eradication

The *K. pneumoniae* ATCC 700603 was tested for biofilm formation in different media as reported before. LB, TSB, TSB + 1% Glucose, M63, and the M63 media were selected for biofilm experiments (20, 30, 31). With some modifications from previous methods, employing CV staining, *K. pneumoniae* cells were grown in M63 salt media at 37°C, 200 rpm shaking conditions overnight. The culture was diluted 1:100 in fresh M63 and dispensed into 96-well tissue culture-treated plates along with different concentrations (1×, 2×, 5×, and 10× MIC) of test compound tridecaptin M and colistin as a positive control. Wells with only media and no culture were taken as a negative control, and the plate was incubated at static conditions for 48 h. Following incubation, the media and planktonic cells were gently removed, and the wells with adhered biofilm were washed three times with 0.9% saline. Subsequently, the formed biofilm was fixed with methanol at room temperature for 15 min and air-dried. The biofilms were then stained with 0.1% CV for 15 min at room temperature. Following staining, wells were washed with 0.9% saline to remove excess dye, and adherent dye was solubilized with 95% ethanol (32). The biofilm inhibition was calculated as the percentage of biofilm remaining after treatment using the formula:


$$Biofilms\;remaining\;(\%)=OD_{595}\;(sample)/OD_{595}\;(control)\times 100\%$$

Similarly, for the biofilm disruption assay, an overnight grown culture was diluted 1:100 and inoculated in a 96-well plate and incubated for 48 h to let the biofilm form. After 48 h, with minimum disturbance, the formed biofilm media is removed. Next, 200 µL of M63 media, with different concentrations as mentioned above of tridecaptin M and colistin, was incubated for another 24 h. Next, the wells are processed to measure the biofilm remaining with crystal violet staining as described for biofilm inhibition. The percentage of biofilm disrupted is calculated as the percentage of biofilm remaining calculated as above subtracted from 100% (33).

### Post-antibiotic effect (PAE)

*K. pneumoniae* ATCC 700603 was cultivated to the mid-exponential phase (OD600nm ≈ 0.3) in CA-MHB. The cultures were then exposed to tridecaptin M at concentrations of ¼ MIC, ½ MIC, and 1 MIC for 2 h at 37°C, 200 rpm. Following treatment, the cultures were centrifuged and resuspended in a fresh medium to remove the compound. To eliminate any residual drug effects, each culture was diluted 100-fold; 200 µL of the diluted culture was added in triplicate to a 96-well plate, and the growth kinetics were performed. An untreated bacterial suspension processed in the same manner served as the control. Absorbance (OD600nm) was measured over 12 h using a microplate reader. The PAE was calculated as: PAE = T50 – C50, where the time required for the treated culture to recover from stress and reach the 50% OD of the maximum OD reached by the control culture, and C50 is the time for the untreated cultures to reach the same absorbance. The OD_600nm_ of all the sets was adjusted to equal before the growth kinetics to lessen the inoculum differences (34). A complementary CFU-based experiment was performed with the same experimental parameters; instead of OD_600nm_, CFU/mL was calculated for up to 10 h, and PAE was calculated as the time difference between the control and treated group to reach 50% growth of the maximum CFU calculated for the control.

### Plasma protein binding

Plasma protein binding was assessed using the rapid equilibrium dialysis (RED) method, utilizing RED insert devices with a dialysis membrane featuring an 8,000 Dalton molecular weight cutoff for proteins. The test/control items in human, rat, and mouse plasma were prepared at a test concentration of 10 µM using 10 mM DMSO stocks, and the samples were added to the plasma chamber of the RED insert. The device was sealed with tape and incubated in a thermomixer at 37°C with shaking at 300 rpm for 4 h. Following incubation, a 50 µL aliquot of plasma and buffer was collected from each insert and diluted with an equal volume of the opposite matrix to mitigate matrix effects. The samples were quantified using LC-MS/MS analysis and were tested in duplicates.

### Metabolic stability

A metabolic stability assay was conducted using human, rat, and mouse liver microsomes for test and control items, both with and without cofactors, in a 24-well plate. For each 100 µL reaction mixture per time point, 45 µL of PBS (50 mM, pH 7.40), 10 µL of a 10× working solution of liver microsomes (5 mg/mL) from human, rat, or mouse, 25 µL of a 4× working solution of test/control items, and 20 µL of a 5× working solution of pre-incubated cofactors were added. For without cofactors, 20 µL of PBS was added instead of pre-incubated cofactors. The reaction mixture was incubated at 37°C for designated time points (with cofactors: 0, 5, 15, 30, 60, and 120 min; without cofactors: 0 and 120 min). At each specified time, a 100 µL sample was taken, processed through a sample extraction procedure, and submitted for LC-MS/MS analysis (35). Half-life (t_1/2_) of test/control items was determined using the following equation


$$t_{1/2}\;(min)=0.693/k$$

where, elimination rate constant (k) = −(slope of linear regression curve).

### hERG channel inhibition assay

The NPC-1 chip is prepared by filling it with 5 µL of an internal buffer (KCl 50 mM, NaCl 10 mM, KFl 60 mM, EGTA 20 mM, Hepes/KOH 10 mM; pH 7.2, osmolarity 285 mOsmol) and secured on the chip holder. A Faraday cage is mounted, and 5 µL of external buffer (NaCl 140 mM, KCl 4 mM, MgCl2 1 mM, CaCl2 2 mM, D-Glucose 5 mM, Hepes/NaOH 10 mM; pH 7.4, osmolarity 298 mOsmol) is added. The experiment starts upon reaching a resistance of 2-3.5 MOhm. A 5 µL cell suspension is added after generating a suction pulse to capture a cell, increasing resistance to 5 MOhm, and triggering the sealing step. Following 2–3 washes, 20 µL of seal enhancer solution (NaCl 80 mM, KCl 3 mM, MgCl2 10 mM, CaCl2 35 mM, Hepes/HCl 10 mM; pH 7.4, osmolarity 298 mOsmol) is added to enhance the seal. A hERG protocol measures ion current every 10 s with voltage steps: holding at −80 mV, depolarizing to +40 mV, and repolarizing to −40 mV. Stable current is achieved in ~2 min. Vehicle control (20 µL) is added, followed by test items (1 µM and 10 µM) after 2–3 washes, with each tested for 3–5 min. Quinidine sulfate verifies system sensitivity. The results include current amplitudes for each concentration and control (36).

### CYP P450 inhibition assay

The CYP inhibition assay was performed for test/control inhibitor items in duplicates using a 96-well fluorescence plate. An example of the preparation of CYP inhibition is given below in Table 8**.**

**TABLE 8:** Preparation of CYP inhibition reaction mixture

Composition	Reaction mixture for one reaction (µL)
Test/ control item (2.5×)	40
CYP P450 Master Premix (2×)	50
Substrate-NADP^+^ Mixture (10×)	10
Total volume	100 µL

The plate with CYP master premix with test/control was incubated to allow the test/control samples to interact with CYP enzyme for 10 ± 1 min at 25°C under shaking conditions (400 rpm) using Thermomixer. The assay was initiated by adding 10 µL of a substrate and NADP^+^ mixture to all wells, including a vehicle control and an assay blank. The plate was then incubated at 25°C for 25 min with shaking at 400 rpm using a thermomixer. After incubation, the reaction was quenched by adding 50 µL of stop reagent (0.5 M Tris base) to each well. Fluorescence was measured using a microplate reader at excitation/emission wavelengths of 415/460 nm, respectively (37).

### AMES test

The *Salmonella typhimurium* histidine-dependent engineered tester strains are allowed to incubate with S9 enzyme mix or PBS (without S9 set) for 30 min at 35°C. After incubation, the mixture was added to 2 mL of top agar tubes maintained at 46 ± 2°C and plated onto minimal glucose agar plates. After solidification, the plates are incubated at 37°C. *Salmonella typhimurium* auxotrophic mutants, if grown on minimal media, are counted as the number of revertant colonies due to the mutagenic potential of the compound tested. Positive controls, growth controls, and sterility checks are included during the experimentation for the sensitivity of the experiment (38).

### Pharmacokinetics in male Sprague–Dawley rats

Male Sprague–Dawley rats, approximately 10 weeks old, were administered tridecaptin M (5 mg/kg) dissolved in normal saline via the tail vein for the IV route (5 mL/kg). Blood samples were collected from the retro-orbital sinus at 0.08, 0.25, 0.5, 1, 2, 4, 6, 8, and 24 h post-administration into tubes containing K2EDTA (2 mg/mL blood) as an anticoagulant. The samples were then centrifuged at 4,000 rpm for 10 min at 4°C to separate plasma, which was stored at −80°C until analysis. Concentration of tridecaptin M was determined by LC-MS/MS. For subcutaneous pharmacokinetics in male Sprague–Dawley rats, tridecaptin M at 5 mg/kg was administered subcutaneously (10 mL/kg). Blood samples were collected at 0.25, 0.5, 1, 2, 4, 8, and 24 h post-administration. The samples were then centrifuged at 10,000 rpm for 10 min at 4°C, and the concentration of tridecaptin M was determined using HPLC analysis.

### Toxicity, histopathology, and blood biochemical studies

#### 14-day repeat dose toxicity

As colistin is known for its nephrotoxic and neurotoxic effects, the maximum dose recommended is 15 mg/kg/day divided into 2–3 doses with a minimum 4 h interval between the doses. Hence, we performed repeated dose toxicity for 14 days of tridecaptin M in comparison to colistin at 15 mg/kg/day and vehicle control, 2.5% DMSO in PBS was used, divided into two doses at an interval of 12 h in 8–10 weeks old female Balb/c mice (n = 6). After 12 h from the last dose, blood samples were drawn, and mice were euthanized to isolate the kidney and liver. Organs were fixed in 10% formalin for histopathology, and the serum was centrifuged from whole blood. The blood was left undisturbed for 20 min and centrifuged at 1,000 × *g* for 10 min to isolate serum for biochemical tests of the kidney and liver toxicity evaluation (22).

#### Acute dose toxicity

Acute toxicity was performed on Balb/c female mice, 8 weeks old. This study was adopted and modified from a study described previously (39). Initial screening was done with 100 mg/kg, 200 mg/kg, 400 mg/kg, and 600 mg/kg doses of tridecaptin M and vehicle control (100% DMSO). Mice were observed for 3 days for any visible signs of reaction, lethargy, or mortality. The highest concentration at which no signs of toxicity were observed was used for a confirmatory test. Eight mice were taken, four in each group (two male and two female), one group was administered vehicle control (DMSO), and the other group was administered 600 mg/kg of tridecaptin M. After 3 days, blood was collected to obtain serum for biochemical tests, and mice were euthanized to remove organs (preserved in 10% formalin) for histopathological analysis. The blood was left undisturbed for 20 min and centrifuged at 1,000 × *g* for 10 min to isolate serum for biochemical tests of the kidney and liver toxicity evaluation.

#### Intravenous acute toxicity

In total, 8–10 week old female Balb/c mice (n = 3) were administered tridecaptin M starting at 15 mg/kg, 25 mg/kg, 50 mg/kg, and 100 mg/kg. Colistin is shown to exhibit high toxicity, ranging from 10 mg/kg to 20 mg/kg. In this study, we administered 18 mg/kg of colistin to mice intravenously. Mice were observed for 7 days for signs of toxicity, and at the end of 7 days, mice that survived were anesthetized, and major organs were collected for histopathological analysis as above. Later, in the second set, intermediate concentrations of 20 mg/kg, 25 mg/kg, 30 mg/kg, 35 mg/kg, and 40 mg/kg doses were also administered to *n* = 4 mice. In these groups, blood was collected at 24 h post administration or immediately after visible movement stopped (40 mg/kg group), serum was separated and pooled together for each group *n* = 4, and biochemical serum tests were conducted.

### *In vivo* efficacy in mouse infection models

#### Blood infection model

In total, 8–10 week-old female Balb/c mice (n = 6) were rendered neutropenic with cyclophosphamide 150 mg/kg, 4 days before and 100 mg/kg 1 day before infection. *K. pneumoniae* AH-16, a colistin-resistant clinical strain, was grown to the exponential phase, and a final OD_600_ of 0.5 was set in PBS; 50 µL of bacterial suspension was administered via the tail vein using a sterile syringe (0.25 × 6 mm). At 4 h post-infection, treatment with colistin and tridecapin M at 10 mg/kg single dose was administered subcutaneously to each group. At this point, the pre-treatment group was processed for blood collection via retro-orbital puncture, serially diluted, and spotted on MHA plates for CFU count to ensure infection establishment. After 20 h post-treatment, the remaining groups were also processed for blood withdrawal, and CFU was determined (40).

#### Mucin peritonitis survival model

The survival study was performed by following a method described earlier, with some modifications; 8–10 week old, female BALB/c mice, 10 mice in each group, were infected with 100μL (OD_600nm_ = 1) of *K. pneumoniae AH-16* bacterial suspension prepared in porcine mucin 5% via intraperitoneal injection. Mice were injected subcutaneously with tridecaptin M (10 mg/kg) and colistin (5 mg/kg). The treatment was given in three doses at 2 h, 12 h, and 24 h post-infection to ensure a 15 mg/kg/day maximum dose limit for colistin. The survival or death of mice was monitored and recorded every 12 h for 7 days (41).

#### Lung infection model

In total, 6- to 8-week-old female BALB/c mice (n = 6) were made neutropenic as mentioned in the above protocol. An inoculum of *K. pneumoniae* ATCC 700603 adjusted to OD_600_ = 1.0 was re-suspended in sterile 1× PBS (42). Infection was induced nasally with a 20µL volume, and treatment of colistin at 15 mg/kg, tridecaptin M at 10 mg/kg, 20 mg/kg, 50 mg/kg, and 100 mg/kg was given 4h after infection via a subcutaneous route. At 4 h pre-treatment, the group was euthanized to confirm CFU before treatment, and 20 h after treatment, the remaining mice (control and treatment group) were euthanized, and the lungs were isolated. Homogenization followed by dilution with PBS was spotted on the MHA plate, and CFU levels before and after treatment were calculated (43).

#### Peritonitis infection model for intravenous efficacy

Balb/c mice (*n* = 6), 8-10 weeks old, are rendered neutropenic with cyclophosphamide as in previous experiments. A bacterial load of *K. pneumoniae* ATCC 700603 standard strain corresponding to ≈10^7^ CFU/mL was used, and 100 μL was administered intraperitoneally to cause multi-organ infection conditions. At 4 h, the pre-treatment group was euthanized, and the right kidney of each mouse was harvested, homogenized, serially diluted, and spotted on MHA plates to determine bacterial CFU count per kidney. Simultaneously, at 4 h, the remaining groups were administered 5 mg/kg colistin, 5 mg/kg tridecaptin M, 10 mg/kg tridecaptin M, and vehicle control (PBS); 24 h after treatment, the remaining groups were euthanized, and the CFU count in the right kidney was determined the same as the 4 h pre-treatment group.

### Statistical analysis

Studies including biological replicates (three or more) are plotted as mean and standard deviation or standard error of mean. Statistical significance and comparison between multiple groups were determined using one-way analysis of variance (ANOVA) or *t*-test with GraphPad Prism 8.0.1 software. *P* values were considered significant when *P < 0.05 and highly significant when **P < 0.01, ***P < 0.001, and ****P < 0.0001.

## SUPPLEMENTAL MATERIAL

Supplemental material aac.01083-25-s0001.docx Fig. S1 to S3; Table S1 to S7.

ASM does not own the copyrights to Supplemental Material that may be linked to, or accessed through, an article. The authors have granted ASM a non-exclusive, world-wide license to publish the Supplemental Material files. Please contact the corresponding author directly for reuse.
