# Supplementary material for: Preclinical evaluation of tridecaptin M: in vitro and in vivo efficacy against colistin-resistant Gram-negative bacterial pathogens and pharmacokinetics
Source: Antimicrob Agents Chemother. 2025 Sep 11;69(10):e01083-25. doi: 10.1128/aac.01083-25 (PMC12486805; doi:10.1128/aac.01083-25)
Supplement: Supplemental material — Fig. S1 to S3; Table S1 to S7. [file aac.01083-25-s0001.docx]

**Supplementary Material**

**Preclinical Evaluation of Tridecaptin M: *In vitro* and *in vivo* Efficacy against Colistin-Resistant Gram-Negative Bacterial Pathogens and Pharmacokinetics**

Vrushali Raka,^a,b^ Manoj Jangra,^a^* Parminder Kaur,^a$^ Rajneesh Dadwal,^a^ Shubhangi Kansal,^c^ Archana Angrup,^c^ Pallab Ray,^c^ Hemraj Nandanwar^a,b^#

^a^Clinical Microbiology & Antimicrobial Research Laboratory, CSIR-Institute of Microbial Technology, Sector 39-A, Chandigarh, India

^b^Academy of Scientific & Innovative Research (AcSIR), Ghaziabad, Uttar Pradesh-201002, India

^c^Department of Medical Microbiology, Post Graduate Institute of Medical Education and Research, Chandigarh, India

**#Address for correspondence**

Dr. Hemraj Nandanwar, Chief Scientist, CSIR-Institute of Microbial Technology, Sector 39-A, Chandigarh, India-160036**. E-mail ID**: [hemraj.nandanwar@csir.res.in](mailto:hemraj.nandanwar@csir.res.in)

**Telephone**: +91-172-2880338 Fax: +91-172-2690585/2690632

*Present Address: Department of Biochemistry and Biomedical Sciences, McMaster University, Hamilton, Ontario, Canada

^$^Present Address: Hematology-Oncology Unit, Department of Pediatrics, Postgraduate Institute of Medical Education and Research, Chandigarh, India


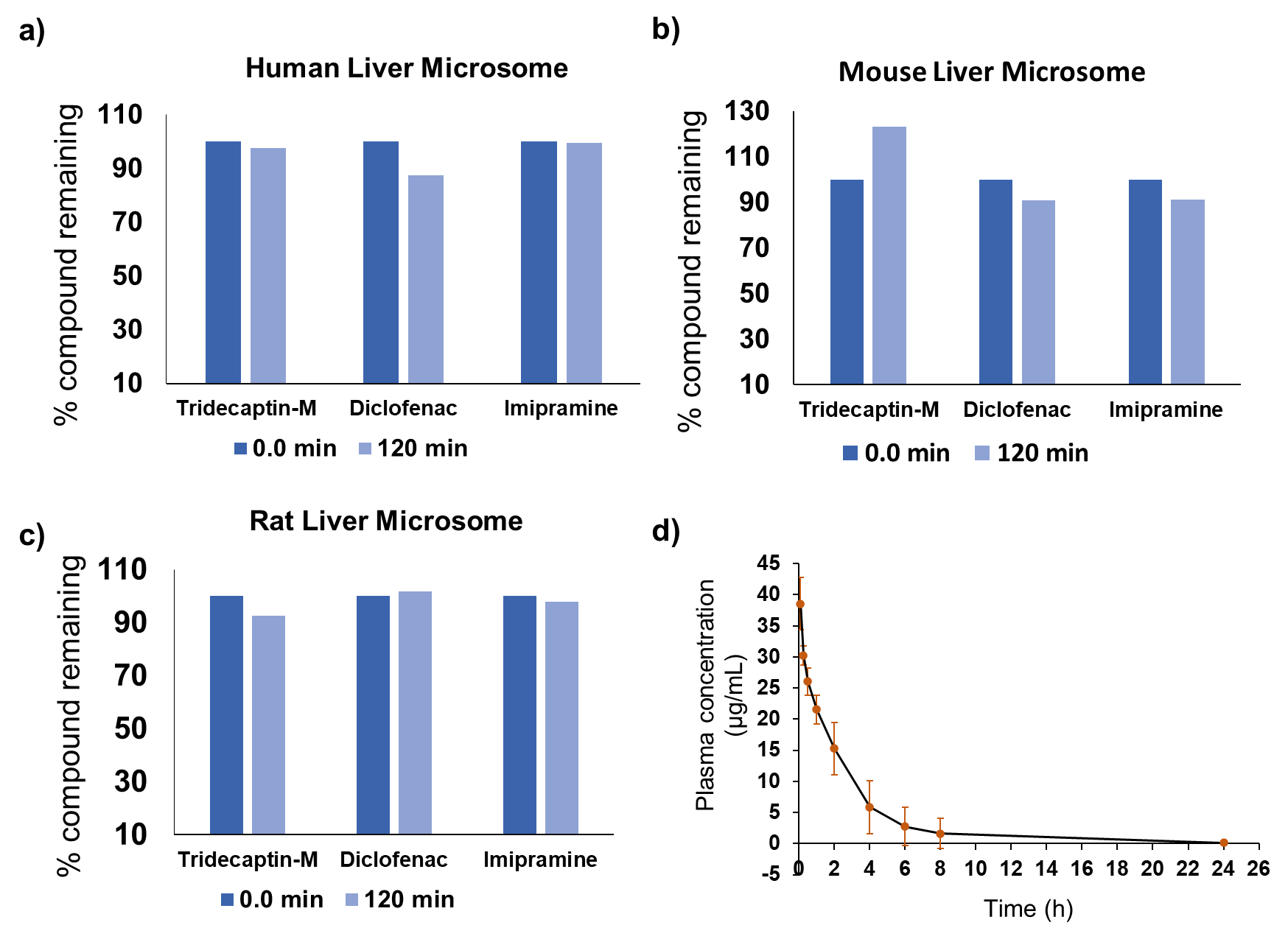


**Figure S1**: Liver Microsomal stability of tridecaptin M and positive controls (diclofenac & imipramine) without cofactors tested at 0 min and 120 min against a) human liver microsome, b) mouse liver microsome and c) rat liver microsome. Values plotted as percent compound remaining as compared to 0 min. The experiment was performed in duplicate. d) Pharmacokinetic plasma concentration in ng/mL after administration of a 5mg/kg b.w dose intravenously in Rat (n=5) was tested at eight time points over 24h. The plasma concentration of tridecaptin M was maintained above MIC (4µg/mL) for about 4h.


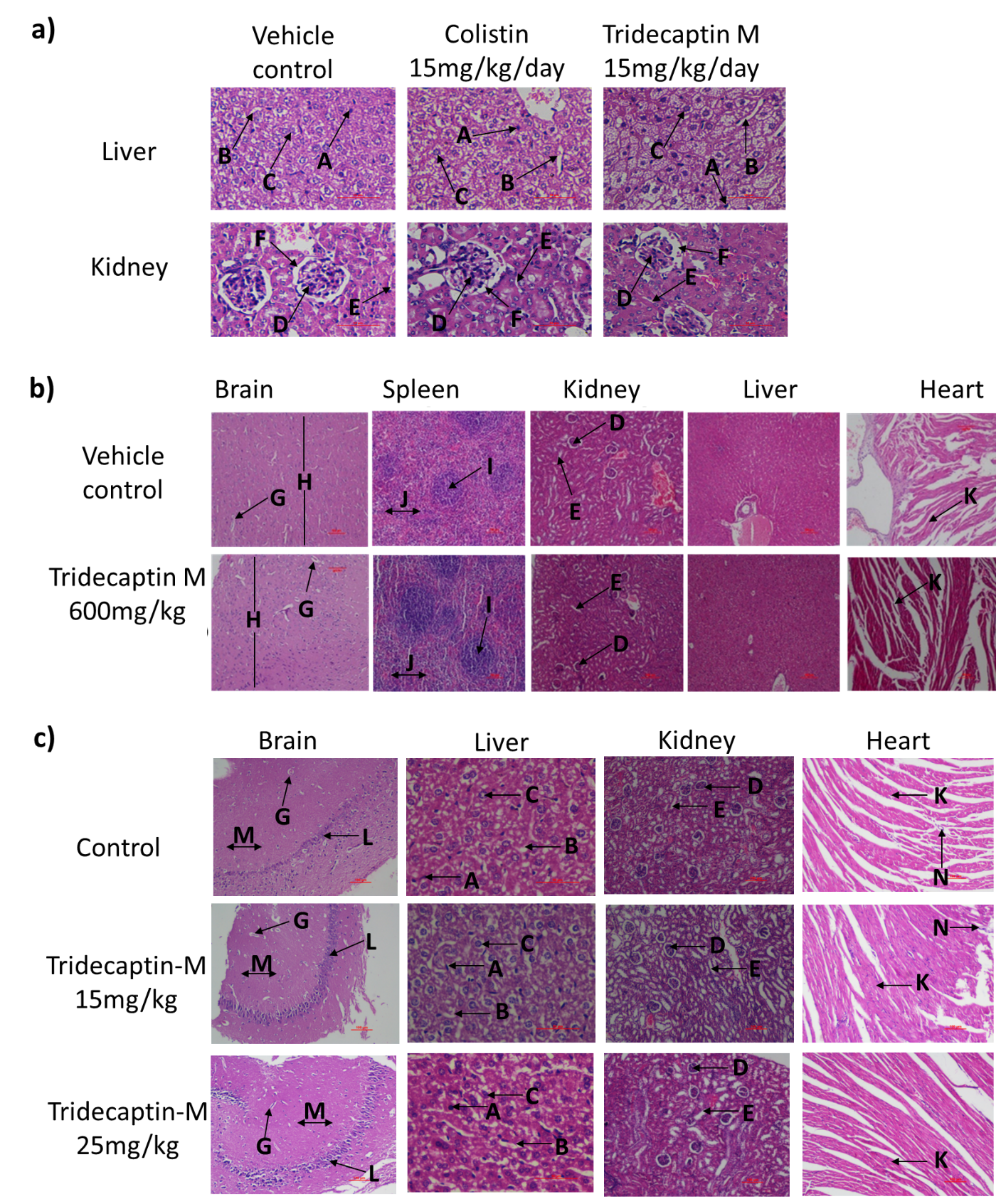


**Figure S2:** a) Histopathological analysis representative images for kidney and liver stained with eosin and hematoxylin from each group administered tridecaptin M (15mg/kg/day), colistin (15mg/kg/day) and control (2.5% DMSO/day) divided into two doses for 14 days (to follow typical 2 week regimen used for colistin), Scale 50 µm. b) Balb/c mice (n=3) were given 100mg/kg, 200mg/kg, 400mg/kg, and 600mg/kg doses of tridecaptin-M to determine the maximum tolerated dose subcutaneously. No mortality was observed; therefore, histopathology analyses of major organs of mice were performed. Vehicle control (DMSO) and Tri-M (tridecaptin M) 600mg/kg group representative images are shown here. Scale 100µm. c) Acute intravenous toxicity - Histopathological analysis of major organs, namely, brain, liver, kidney, and heart, stained with eosin and hematoxylin after administration of tridecaptin M at 15mg/kg and 25mg/kg single dose intravenously (n=3). Mice were observed for 3 days before harvesting organs. Scale 100 µm for the brain, kidney, heart, and 50µm liver. No significant changes in histopathological analysis were observed for all three experiments. (A: Kupffer cells, B: Sinusoids, C: Hepatocytes, D: Glomerulus, E: Tubules, F: Bowman’s space, G: Blood vessel, H: Cerebral cortex, I: White pulp, J: Red pulp, K: Muscle fibre, L: Collection of neurons, M: Glial tissue, N: Capillary)


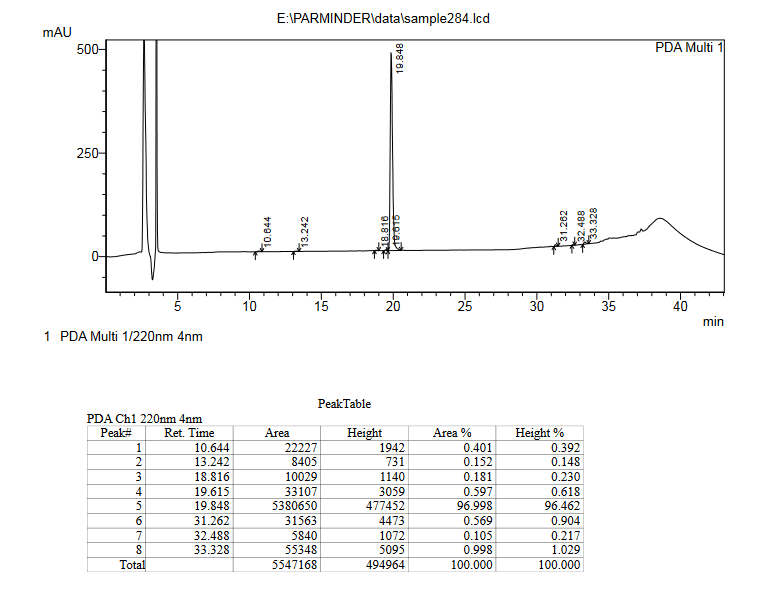


**Figure S3:** Purity check by HPLC after purification and lyophilization of tridecaptin-M

**Table S1**: Microsomal stability in Mouse, Rat, and Human Liver microsomes (% inhibition)

| Test/Control item | Liver Microsome | 0.0 min | 15 min | 30 min | 60 min | 120 min | Half-life (min) | CL (µL/min/mg) |
| --- | --- | --- | --- | --- | --- | --- | --- | --- |
| **Tridecaptin M** | Mouse | 100.00 | 82.38 | 83.79 | 88.10 | 74.71 | >120 | <11.55 |
|  | Rat | 100.00 | 86.42 | 76.55 | 70.06 | 58.76 | >120 | <11.55 |
|  | Human | 100.00 | 57.96 | 50.22 | 53.72 | 48.35 | 78.75 | 17.60 |
| **Diclofenac** | Mouse | 100.00 | 85.30 | 77.65 | 58.74 | 40.13 | 92.40 | 15.00 |
|  | Rat | 100.00 | 102.27 | 88.60 | 67.16 | 64.55 | >120 | <11.55 |
|  | Human | 100.00 | 53.23 | 36.48 | 11.58 | 8.30 | 19.63 | 70.60 |
| **Imipramine** | Mouse | 100.00 | 4.58 | 0.55 | 0.10 | 0.05 | 12.27 | 113.00 |
|  | Rat | 100.00 | 77.20 | 49.16 | 26.02 | 11.42 | 37.87 | 36.60 |
|  | Human | 100.00 | 86.77 | 76.59 | 74.19 | 71.63 | >120 | <11.55 |

**Table S2:** CYP 1A2 inhibition by furafylline (positive control) and tridecaptin M

| \| **Test concentration µM** \| **% Inhibition by Furafylline** \| \| --- \| --- \| \| 30 \| 95.63 \| \| 10 \| 90.81 \| \| 3 \| 67.71 \| \| 1 \| 45.31 \| \| 0.3 \| 9.30 \| \| 0.1 \| < 0 \| \| 0.03 \| < 0 \| \| 0.01 \| < 0 \| | \| **Test concentration µM (µg)** \| **% inhibition by Tridecaptin M** \| \| --- \| --- \| \| 30 (44.6) \| < 0 \| \| 10 (14.8) \| < 0 \| \| 3 (4.4) \| < 0 \| \| 1 (1.4) \| < 0 \| \| 0.3 (0.46) \| < 0 \| \| 0.1 (0.14) \| < 0 \| \| 0.03 (0.04) \| < 0 \| \| 0.01 (0.01) \| < 0 \| |
| --- | --- | --- | --- | --- | --- | --- | --- | --- | --- | --- | --- | --- | --- | --- | --- | --- | --- | --- | --- | --- | --- | --- | --- | --- | --- | --- | --- | --- | --- | --- | --- | --- | --- | --- | --- | --- | --- |

**Table S3:** CYP 2D6 inhibition by quinidine (positive control) and tridecaptin M

| \| **Test concentration µM** \| **% Inhibition by Quinidine** \| \| --- \| --- \| \| 1 \| 95.71 \| \| 0.3 \| 92.48 \| \| 0.1 \| 93.42 \| \| 0.03 \| 92.59 \| \| 0.01 \| 88.62 \| \| 0.003 \| 52.41 \| \| 0.001 \| 13.98 \| \| 0.0003 \| < 0 \| | \| **Test concentration µM (µg)** \| **% inhibition by Tridecaptin M** \| \| --- \| --- \| \| 30 (44.6) \| 13.11 \| \| 10 (14.8) \| 10.95 \| \| 3 (4.4) \| 1.28 \| \| 1 (1.4) \| < 0 \| \| 0.3 (0.46) \| < 0 \| \| 0.1 (0.14) \| < 0 \| \| 0.03 (0.04) \| < 0 \| \| 0.01 (0.01) \| < 0 \| |
| --- | --- | --- | --- | --- | --- | --- | --- | --- | --- | --- | --- | --- | --- | --- | --- | --- | --- | --- | --- | --- | --- | --- | --- | --- | --- | --- | --- | --- | --- | --- | --- | --- | --- | --- | --- | --- | --- |

**Table S4:** CYP 2C9 inhibition by Sulfaphenazole (positive control) and tridecaptin M

| \| **Test concentration µM** \| **% Inhibition by**  **Sulfaphenazole** \| \| --- \| --- \| \| 3 \| 57.87 \| \| 1 \| 53.46 \| \| 0.3 \| 45.25 \| \| 0.1 \| 34.72 \| \| 0.03 \| 27.18 \| \| 0.01 \| 11.27 \| \| 0.003 \| 3.42 \| \| 0.001 \| < 0 \| | \| **Test concentration µM (µg)** \| **% inhibition by Tridecaptin M** \| \| --- \| --- \| \| 30 (44.6) \| 25.60 \| \| 10 (14.8) \| 0.82 \| \| 3 (4.4) \| < 0 \| \| 1 (1.4) \| 0.05 \| \| 0.3 (0.46) \| < 0 \| \| 0.1 (0.14) \| < 0 \| \| 0.03 (0.04) \| 0.93 \| \| 0.01 (0.01) \| < 0 \| |
| --- | --- | --- | --- | --- | --- | --- | --- | --- | --- | --- | --- | --- | --- | --- | --- | --- | --- | --- | --- | --- | --- | --- | --- | --- | --- | --- | --- | --- | --- | --- | --- | --- | --- | --- | --- | --- | --- |

**Table S5:** CYP 2C19 inhibition by Tranylcypromine (positive control) and tridecaptin M

| \| **Test concentration µM** \| **% Inhibition by**  **Tranylcypromine** \| \| --- \| --- \| \| 50 \| 95.05 \| \| 20 \| 88.59 \| \| 5 \| 72.76 \| \| 2 \| 56.17 \| \| 0.5 \| 25.87 \| \| 0.2 \| 13.43 \| \| 0.05 \| 7.06 \| \| 0.02 \| 2.83 \| | \| **Test concentration µM (µg)** \| **% inhibition by Tridecaptin M** \| \| --- \| --- \| \| 30 (44.6) \| 11.65 \| \| 10 (14.8) \| 5.67 \| \| 3 (4.4) \| 0.13 \| \| 1 (1.4) \| < 0 \| \| 0.3 (0.46) \| < 0 \| \| 0.1 (0.14) \| < 0 \| \| 0.03 (0.04) \| < 0 \| \| 0.01 (0.01) \| < 0 \| |
| --- | --- | --- | --- | --- | --- | --- | --- | --- | --- | --- | --- | --- | --- | --- | --- | --- | --- | --- | --- | --- | --- | --- | --- | --- | --- | --- | --- | --- | --- | --- | --- | --- | --- | --- | --- | --- | --- |

**Table S6:** CYP 3A4 inhibition by Ketoconazole (positive control) and tridecaptin M

| \| **Test concentration µM** \| **% Inhibition by Ketoconazole** \| \| --- \| --- \| \| 10 \| 98.46 \| \| 3 \| 97.77 \| \| 1 \| 96.03 \| \| 0.3 \| 87.40 \| \| 0.1 \| 85.08 \| \| 0.03 \| 56.68 \| \| 0.01 \| 39.19 \| \| 0.003 \| < 0 \| | \| **Test concentration µM (µg)** \| **% inhibition by Tridecaptin M** \| \| --- \| --- \| \| 30 (44.6) \| 26.59 \| \| 10 (14.8) \| 27.98 \| \| 3 (4.4) \| 9.00 \| \| 1 (1.4) \| 3.77 \| \| 0.3 (0.46) \| < 0 \| \| 0.1 (0.14) \| < 0 \| \| 0.03 (0.04) \| < 0 \| \| 0.01 (0.01) \| < 0 \| |
| --- | --- | --- | --- | --- | --- | --- | --- | --- | --- | --- | --- | --- | --- | --- | --- | --- | --- | --- | --- | --- | --- | --- | --- | --- | --- | --- | --- | --- | --- | --- | --- | --- | --- | --- | --- | --- | --- |

**Table S7:** AMES test to check the mutagenic effect of tridecaptin M in a concentration-dependent manner.

| **Test Item**  **(μg /plate)** | ***Salmonella typhimurium*** | | | | | **Remark** |
| --- | --- | --- | --- | --- | --- | --- |
|  | **TA102** | **TA100** | **TA98** | **TA1537** | **TA1535** |  |
| **0.03** | NC, NR | NC, NR | NC, NR | NC, NR | NC, NR | Mutagenic response not observed |
| **0.10** | NC, NR | NC, NR | NC, NR | NC, NR | NC, NR | Mutagenic response not observed |
| **0.32** | NC, NR | NC, NR | NC, NR | NC, NR | NC, NR | Mutagenic response not observed |
| **1.00** | NC, NR | NC, NR | NC, NR | NC, NR | NC, NR | Mutagenic response not observed |
| **3.16** | NC, NR | NC, NR | NC, NR | NC, NR | NC, NR | Mutagenic response not observed |
| **10** | PI, RC | PI, RC | PI, RC | PI, RC | PI, RC | Cytotoxicity observed |
| **Basal control** | NC, NR | NC, NR | NC, NR | NC, NR | NC, NR | Mutagenic response not observed |
| **Positive Control** | RO | RO | RO | RO | RO | Mutagenic response observed |

NC: No Cytotoxicity, NR: No Revertant, PI: Partial Inhibition of (due to cytotoxic effect of tridecaptin M on *Salmonella typhimurium*), RC: Reduced colonies, RO: Revertant Observed
